# Innate immune activation of astrocytes impairs neurodevelopment via upregulation of follistatin-like 1 and interferon-induced transmembrane protein 3

**DOI:** 10.1186/s12974-018-1332-0

**Published:** 2018-10-22

**Authors:** Shinnosuke Yamada, Norimichi Itoh, Taku Nagai, Tsuyoshi Nakai, Daisuke Ibi, Akira Nakajima, Toshitaka Nabeshima, Kiyofumi Yamada

**Affiliations:** 1 Department of Neuropsychopharmacology and Hospital Pharmacy, Nagoya University Graduate School of Medicine, 65 Turumai-cho, Showa-ku, Nagoya, Aichi 466-8560 Japan; 2 Department of Chemical Pharmacology, Faculty of Pharmaceutical Science, Meijo University, 150 Yagotoyama, Tenpaku-ku, Nagoya, Japan; 3 Faculty of Agriculture and Life Science, Hirosaki University, 3 Bunkyo-cho, Hirosaki, Aomori 036-8561 Japan; 4 Advanced Diagnostic System Research Laboratory, Fujita Health University, Graduate School of Health Science and Aino University, 1-98 Dengakugakubo, Kutsukake-cho, Toyoake, Aichi 470-1192 Japan

**Keywords:** Astrocyte, Fstl1, Ifitm3, Immune response, Neuron, polyI:C, Schizophrenia, Viral infection

## Abstract

**Background:**

Polyriboinosinic-polyribocytidylic acid (polyI:C) triggers a strong innate immune response that mimics immune activation by viral infections. Induction of interferon-induced transmembrane protein 3 (Ifitm3) in astrocytes has a crucial role in polyI:C-induced neurodevelopmental abnormalities. Through a quantitative proteomic screen, we previously identified candidate astroglial factors, such as matrix metalloproteinase-3 (Mmp3) and follistatin-like 1 (Fstl1), in polyl:C-induced neurodevelopmental impairment. Here, we characterized the Ifitm3-dependent inflammatory processes focusing on astrocyte-derived Fstl1 following polyI:C treatment to assess the neuropathologic role of Fstl1.

**Methods:**

Astrocytes were treated with PBS (control) or polyI:C (10 μg/mL). The conditioned medium was collected 24 h after the polyI:C treatment and used as astrocyte condition medium (ACM). The expression of Fstl1 mRNA and extracellular Fstl1 protein levels were analyzed by quantitative PCR and western blotting, respectively. For functional studies, neurons were treated with ACM and the effects of ACM on dendritic elongation were assayed. To examine the role of Fstl1, recombinant Fstl1 protein and siRNA for Fstl1 were used. To investigate the expression of Fstl1 in vivo, neonatal mice were treated with vehicle or polyI:C on postnatal day 2 to 6.

**Results:**

ACM prepared with polyI:C (polyI:C ACM) contained significantly higher Fstl1 protein than control ACM, but no increase in Fstl1 was observed in polyI:C ACM derived from Ifitm3-deficient astrocytes. We found that the production of Fstl1 involves the inflammatory responsive molecule Ifitm3 in astrocytes and influences neuronal differentiation. In agreement, the levels of Fstl1 increased in the hippocampus of polyI:C-treated neonatal mice. COS7 cells co-transfected with both Fstl1 and Ifitm3 had higher extracellular levels of Fstl1 than the cells transfected with Fstl1 alone. Treatment of primary cultured hippocampal neurons with recombinant Fstl1 impaired dendritic elongation, and the deleterious effect of polyI:C ACM on dendritic elongation was attenuated by knockdown of Fstl1 in astrocytes.

**Conclusions:**

The extracellular level of Fstl1 is regulated by Ifitm3 in astrocytes, which could be involved in polyI:C-induced neurodevelopmental impairment.

**Electronic supplementary material:**

The online version of this article (10.1186/s12974-018-1332-0) contains supplementary material, which is available to authorized users.

## Background

Immune activation in the CNS is associated with the pathophysiology and/or etiology of psychiatric disorders. For example, inflammatory cytokine levels are altered in the serum of schizophrenia patients [1, 2]. Pro- and anti-inflammatory cytokines in patients with major depressive disorders are inversely correlated with severity and symptoms of major depression [3]. Epidemiological evidence also indicates that the risk of schizophrenia increases in offspring born from mothers infected during pregnancy [4–6]. Although much evidence suggests that reactive astrocytes induced by inflammatory cytokines are involved in neurodevelopmental disorders [7, 8], the underlying molecular mechanism is largely unknown.

Interferon-induced transmembrane protein 3 (Ifitm3) is induced by inflammatory cytokines such as interferon-β, interleukin (IL)-6, and tumor necrosis factor (TNF)-α. Ifitm3 acts as an antiviral factor by restricting virus entry [9–12]. Recent studies have indicated that Ifitm3 expression is increased in patients with schizophrenia [13–15]. Previously, we have reported that Ifitm3 expression in astrocytes is associated with neurodevelopmental impairment and contributes to brain dysfunction in mice that received daily polyI:C injection from postnatal day 2 to day 6 [16]. We also found that humoral factors released from polyI:C-treated astrocytes have crucial roles in Ifitm3-dependent impairment of neuronal maturation of cultured neurons [17]. For instance, matrix metalloprotease-3 (Mmp3) is released by polyI:C-treated astrocytes and impairs neuronal maturation [18]. In addition to Mmp3, we have identified several candidates of humoral factors including follistatin like-1 (Fstl1) by proteomic analysis [18].

To investigate the regulation of Fstl1 production and release, we monitored extracellular levels of Fstl1 protein in astrocyte cultures, which were stimulated with polyI:C or prepared from Ifitm3 knockout (KO) mice. We found that an immune activation induced by polyI:C upregulates Fstl1 mRNA expression in astrocytes, and that polyI:C-treated astrocytes have increased extracellular protein levels of Fstl1. Notably, in astrocytes from Ifitm3 KO mice, polyI:C treatment failed to increase the extracellular Fstl1 level. We also demonstrated that recombinant Fstl1 impaired dendritic neurite outgrowth of cultured hippocampal neurons, but knockdown of Fstl1 partially rescued polyI:C-induced impairment of neurite elongation. These results suggest that Fstl1 has an important role in Ifitm3-dependent neuronal impairment.

## Methods

### Animals

C57BL/6J mice were purchased from Japan SLC Inc. (Hamamatsu, Japan). Homozygous *Ifitm3*^*−/−*^ [Ifitm3 KO] mice were generated and characterized as described previously [19]. The animals had free access to food (CE-2, Clea Japan, Tokyo, Japan) and water and were kept under controlled conditions (23 ± 1 °C) with a constant light-dark cycle (light 9:00–21:00). All animals were handled in accordance with the guidelines established by the Institutional Animal Care and Use Committee of Nagoya University, the Guiding Principles for the Care and Use of Laboratory Animals approved by the Japanese Pharmacological Society, and the National Institutes of Health Guide for the Care and Use of Laboratory Animals.

### Astrocyte culture and astrocyte-conditioned medium (ACM) preparation

Secondary astrocyte cultures were prepared as described previously [18]. Briefly, cortices and hippocampi of neonatal mice at postnatal day (PD) 1–2 were mechanically dissociated and digested with 0.3% dispase (Roche Diagnostics GmbH, Mannheim, Germany) and 0.4% DNase (Roche Diagnostics GmbH). The cells were suspended in Dulbecco’s modified Eagle’s medium (DMEM, Sigma-Aldrich, St. Louis, MO) containing 10% fetal bovine serum (FBS, Gibco-BRL, Gaithersburg, MD) and filtered through a 40 μm nylon cell strainer (Falcon-Becton Dickinson, Le Pont de Claix, France). The cell suspension was cultured in a T150 flask at a density of six neonate brains per flask at 37 °C with 5% CO_2_. Confluent primary astrocyte cultures were purified by shaking, plated onto 6-well plates, and grown to confluence in all experiments. Under these conditions, more than 95% of cells were glial fibrillary acidic protein (GFAP)-positive (a marker for astrocytes) and negative for tau/MAP2 and CD11b (neuronal and microglial markers, respectively). Culture medium was replaced with Neurobasal Medium (Invitrogen, Eugene, OR) supplemented with B-27 (Invitrogen) and 1 mM glutamine (Sigma-Aldrich) 6 days before treatment with polyI:C (Sigma-Aldrich). This medium was replaced 3 days before and 24 h before treatment with polyI:C. For western blotting, B-27 was excluded from the last medium. Astrocytes were treated with PBS (control) or polyI:C (10 μg/mL), and conditioned medium was collected 24 h after the polyI:C treatment. For the time course analysis, conditioned medium was collected 6 h and 12 h after polyI:C treatment. Conditioned media were centrifuged at 1000×*g* for 10 min at 4 °C, and the supernatants were used as ACM.

### Microglia culture and microglia-conditioned medium (MCM) preparation

2 weeks after seeding primary astrocyte cultures, microglia were separated from the underlying astrocytic monolayer by shaking for 3 h at 150 rpm. The supernatants, including floating microglia, were centrifuged at 1000 rpm for 10 min and the pellet was resuspended in fresh culture medium. Microglia were plated at 1 × 10^6^ cells/well in a 6-well plate and culture medium was replaced with neurobasal medium supplemented with 1 mM glutamine 1 h after plating. The microglial cultures were > 98% pure as assessed by immunocytochemistry with an anti-Iba1 antibody (Wako, Osaka, Japan) in combination with an anti-GFAP antibody (Sigma-Aldrich) as markers for microglia and astrocytes, respectively. PBS (control) or polyI:C (10 μg/mL) were added 24 h after medium change, and conditioned media were collected 24 h after polyI:C treatment. Conditioned media were centrifuged at 1000×*g* for 10 min at 4 °C, and the supernatants were used as MCM.

### Fstl1 and Ifitm3 transfection for COS7 cells

Myc-tagged mouse Fstl1 expression vector was purchased from OriGene Technologies (Rockville, MD), and myc-tagged mouse Ifitm3 vector was generated as described previously [17]. Control (mock) vector was generated by the removal of cDNA sequence from the cloning site of the expression vector using the appropriate restriction enzyme, then blunt-ended and ligated. The day before transfection, trypsinized COS7 cells were plated at 40 × 10^4^ cells per well (6-well plate). Each well was transfected with 1.25 μg myc-tagged mouse Ifitm3 and Fstl1 or control vectors using Lipofectamine LTX (Invitrogen) and Opti-MEM I Reduced Serum Medium (Invitrogen). 6 h after transfection, growth medium was replaced with 2 mL of MEM without FBS and antibiotics. Conditioned medium and cell lysate samples for western blotting were prepared 30 h after transfection as described in the “Astrocyte culture and astrocyte-conditioned medium (ACM) preparation” section. Recombinant mouse Fstl1 (rmFstl1) was prepared from conditioned medium after transfection of 2.5 μg myc-tagged mouse Fstl1 expression vector. 30 h after transfection, secreted Fstl1 was purified using c-myc tagged Protein Mild Purification Kit ver.2 (MBL, Nagoya, Japan) according to the manufacturer’s instructions. Protein concentration was determined using a protein assay kit (Bio-Rad Laboratories, Hercules, CA, USA).

### Primary cultured neurons and ACM treatment

Primary cultured hippocampal neurons were prepared from C57BL/6J mice on gestational day 15–16 as described previously [18]. Briefly, embryo hippocampi were trypsinized (with 0.25% trypsin and 0.01% DNase) followed by trituration and seeded on coverslips precoated with 0.1 mg/mL poly-D-lysine at a low density (1.0 × 10^4^ cells/well in a 24-well plate). Cells were cultured in Neurobasal Medium with B-27 and 1 mM glutamine. The medium was replaced with polyI:C ACM or control ACM and supplemented with 0.75 μm cytosine β-D-arabinofuranoside (Ara-C, Sigma-Aldrich) on DIV2. For functional studies, rmFstl1 was added to control ACM or culture medium (Neurobasal Medium supplemented with B-27 and 1 mM glutamine) at the final concentrations of 10 nM, 50 nM, 100 nM, or 300 nM on DIV2. The effects of ACM on dendritic elongation were assayed on DIV7. More than 99% pure neurons, as evaluated by anti-tau or anti-MAP2 immunostaining, were obtained from this preparation.

### Knockdown assay

siRNA transfection was performed 6 h before the last medium change. Astrocytes were transfected with Stealth siRNA for Fstl1 (#1 sense: GCCCAGUUGUCUGCUAUCAAGCUAA, #1 antisense: UUAGCUUGAUAGCAGACAACUGGGC; #2 sense: CCUAGACAAGUACUUUAAGAGCUUU, #2 antisense: AAAGCUCUUAAAGUACUUGUCUAGG) or Stealth RNAi siRNA negative control (control siRNA) using Lipofectamine RNAiMAX transfection reagent (all from Invitrogen).

### Western blotting

ACM and MCM were concentrated using Vivaspin 2 Hydrosart 5000 MWCO (Sartorius Stedim Biotech GmbH). After removing the conditioned medium, the remaining cells were washed with ice-cold PBS and collected in lysis buffer [20 mM Tris-HCl (pH 7.4), 150 mM NaCl, 50 mM NaF, 2 mM EDTA, 1% Triton X-100, 1 mM sodium orthovanadate, 0.1% SDS, 1% sodium deoxycholate and protease inhibitor cocktail (Sigma-Aldrich)]. Protein lysates were centrifuged at 15,000×*g* for 20 min. ACM or cell lysates were denatured in Laemmli sample buffer containing 20% β-mercaptoethanol at 95 °C for 5 min. An equal amount of protein for each sample was separated by 10% SDS-PAGE and transferred to a polyvinylidene fluoride (PVDF) membrane (Millipore). The membrane was blocked with detector block solution (KPL, Gaithersburg, MD). The membrane was incubated with goat anti-Fstl1 antibody (R&D Systems, Minneapolis, MN) or goat anti-actin antibody (Santa Cruz Biotechnology, Santa Cruz, CA) at 4 °C overnight. After incubation with horseradish peroxidase-conjugated secondary anti-goat antibody (R&D Systems) for 2 h, the membrane was incubated with ECL prime western blotting detection reagents (GE Healthcare) and protein bands were detected using a luminescent image analyzer (Atto, Tokyo, Japan).

### Total RNA isolation and real-time RT-PCR

After removing the conditioned medium, total RNA of astrocytes and microglia were prepared using RNeasy Mini Kit (Qiagen, Hilden, Germany) and converted into complementary DNA (cDNA) using the SuperScript III First-Strand Synthesis Kit (Invitrogen). Quantitative real-time PCR was performed on a 7300 Real-Time PCR System (Applied Biosystems, Foster City, CA) using Power SYBR Green Master Mix (Applied Biosystems) according to the manufacturer’s protocol. The primers used were as follows: forward, GCCTATGCCTACTCCGTGAAGT and reverse, GCCTGGGCTCCAGTCACAT for Ifitm3; forward, CACCAGGGCACAGCAGAAA and reverse, GTGCTCTGTGCCTCTTCTTAGATCT for Fstl1; and forward, CGATGCCCTGAGGCTCTTT and reverse, TGGATGCCACAGGATTCCA for β-actin used as an internal control. Real-time PCR reactions were conducted as follows: initial 2 min incubation at 50 °C and 10 min incubation at 95 °C, followed by 40 reaction cycles of 95 °C for 15 s and 60 °C for 1 min. Fluorescent signals were monitored at the extension step of 60 °C in each cycle. For each sample test, each PCR reaction had two replicates and the relative gene expression differences were quantified using the comparative Ct method (^ΔΔ^Ct).

### Immunocytochemistry

Cells were fixed in 4% paraformaldehyde in 0.1 M phosphate buffer (pH 7.4) for 20 min and then permeabilized with 0.1% Triton X-100 for 10 min. After incubation in blocking solution (1% goat and 1% donkey serum in PBS) for 30 min, mouse anti-tau (1:500, Santa Cruz Biotechnology) and rabbit anti-MAP2 (1:1000, Millipore) antibodies diluted in blocking solution were added to the cells. After overnight incubation with primary antibodies at 4 °C, the cells were treated with goat anti-mouse Alexa Fluor (AF) 488 and anti-rabbit AF568 antibodies (1:1,000, Invitrogen) for 2 h at room temperature. The cells were mounted in fluorescence mounting medium (Dako, Glostrup, Denmark) and photographed under a fluorescence microscope (Zeiss, Jena, Germany) using AxioCam MRc5 (Zeiss).

### Dendritic elongation assay

Dendritic elongation of cultured hippocampal neurons was analyzed in accordance with a previous study [18]. Axons were identified by double immunostaining in terms of tau-positive (axonal marker) and MAP2-negative (dendritic marker), and only MAP2-positive neurites were defined as dendrites. Neurons that clearly had tau- or MAP2-positive neurites were selected randomly by an expert researcher who was blinded to the experimental groups. Dendrites were traced automatically with the same configuration using Neurolucida software (MicroBrightField, Williston, VT) and total dendritic length in a single neuron was calculated using Neuroexplorer (MicroBrightField). This assay was performed for three independent experiments.

### Immunohistochemistry (IHC) for polyI:C-treated neonatal mice

All litters from C57BL/6J mice were randomly divided into two groups: vehicle- and polyI:C-treated. Neonatal C57BL/6J mice were administered with a daily subcutaneous injection of saline (control) or polyI:C (5 mg/kg, Sigma-Aldrich) between PD2 and PD6. Immunohistochemistry was conducted as described previously [17]. Neonatal mice were deeply anesthetized with diethyl ether 24 h after the final polyI:C treatment and perfused transcardially with saline, followed by 4% paraformaldehyde in phosphate-buffered saline (PBS, pH 7.4). The brains were removed and cryoprotected. 20-μm-thick coronal brain sections were cut on a cryostat and mounted on slides. The sections were denatured in a microwave oven in 0.01 M citrate buffer (pH 6.0). After blocking with 5% donkey and 5% goat serum/PBS, mouse anti-glial fibrillary acidic protein (GFAP, a marker for astrocytes, 1:1,000, Sigma-Aldrich) and rat anti-Fstl1 (1:100, R&D systems) were added to the sections. After washing in PBS, goat anti-mouse Alexa Fluor (AF) 568 and anti-rat AF488 antibodies (1:1,000, Invitrogen) were added to the sections. The samples were observed using a confocal-laser scanning microscope (LSM 700 Axio Imager; Zeiss).

### Statistical analysis

Data are shown as the mean ± SE. Differences between two groups were analyzed by two-tailed Student’s *t* test and the data distribution was tested for normality with Shapiro-Wilk test. One-way and two-way analyses of variance (ANOVA) followed by Bonferroni post hoc test was applied for differences in three or more groups.

## Results

**Fig. 1:**
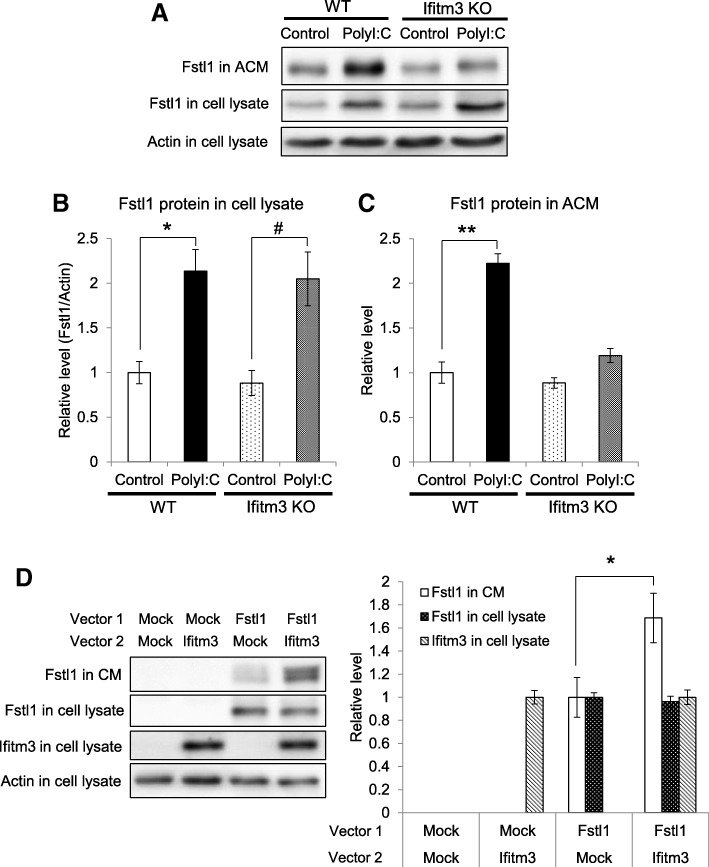
Ifitm3 regulates the extracellular level of Fstl1. **a** Representative western blot images of Fstl1 protein expression in cell lysates and ACM derived from WT and Ifitm3 KO astrocytes 24 h after polyI:C treatment. **b**, **c** Fstl1 protein levels in cell lysates (**b**) and ACM (**c**) derived from WT and Ifitm3 KO astrocytes after polyI:C treatment. Values indicate the means ± SE (*n* = 3). *p < 0.05, ^#^*p* < 0.05, and **p < 0.01 versus the respective control treatment. **d** The extracellular level of Fstl1 in conditioned medium after Fstl1 and Ifitm3 overexpression. COS7 cells were co-transfected with Fstl1, Ifitm3, and control vectors (mock) as indicated respectively. Overexpression of Ifitm3 increased Fstl1 protein in CM without affecting Fstl1 expression in COS7 cell lysates. Data show the mean ± SE (*n* = 6). *p < 0.05 versus Fstl1/mock. CM, conditioned medium

We previously reported that Fstl1 was a candidate molecule responsible for polyI:C-induced neurodevelopmental impairment, and that depletion of Ifitm3 in astrocytes attenuated polyI:C ACM-induced neurodevelopmental impairment [17, 18]. We compared changes in Fstl1 protein levels between WT and Ifitm3 KO astrocytes after polyI:C treatment (Fig. 1a). Two-way ANOVA revealed significant main effects of polyI:C treatment on Fstl1 protein levels in both cell lysates (polyI:C treatment: *F*(1,8) = 28.80, *p* < 0.01; genotype: *F*(1,8) = 0.22, *p* = 0.65; interaction of polyI:C treatment and genotype: *F*(1,8) = 0.01, *p* = 0.94, Fig. 1b) and ACM (polyI:C treatment: *F*(1,8) = 66.09, *p* < 0.01; genotype: *F*(1,8) = 37.21, *p* < 0.01); with an interaction of polyI:C treatment and genotype: (*F*(1,8) = 23.77, *p* < 0.01, Fig. 1c). PolyI:C treatment significantly increased Fstl1 protein levels in the cell lysates of both WT and Ifitm3 KO astrocytes (*p* < 0.05) (Fig. 1b). Notably, polyI:C treatment significantly increased Fstl1 levels in ACM from WT astrocytes but had no effect on extracellular Fstl1 levels in ACM from Ifitm3 KO astrocytes (Fig. 1c). To examine the influence of Ifitm3 expression on extracellular Fstl1 levels, COS7 cells were co-transfected with Fstl1 and Ifitm3. Fstl1 in COS7 cell lysates was not affected by overexpression of Ifitm3. On the other hand, Fstl1 levels in conditioned medium (CM) from COS7 cells co-transfected with Fstl1 and Ifitm3 were markedly higher than the levels from Fstl1 alone transfection (*p* < 0.05, Fig. 1d). These results suggest that the Fstl1 release is regulated by Ifitm3.

**Fig. 2:**
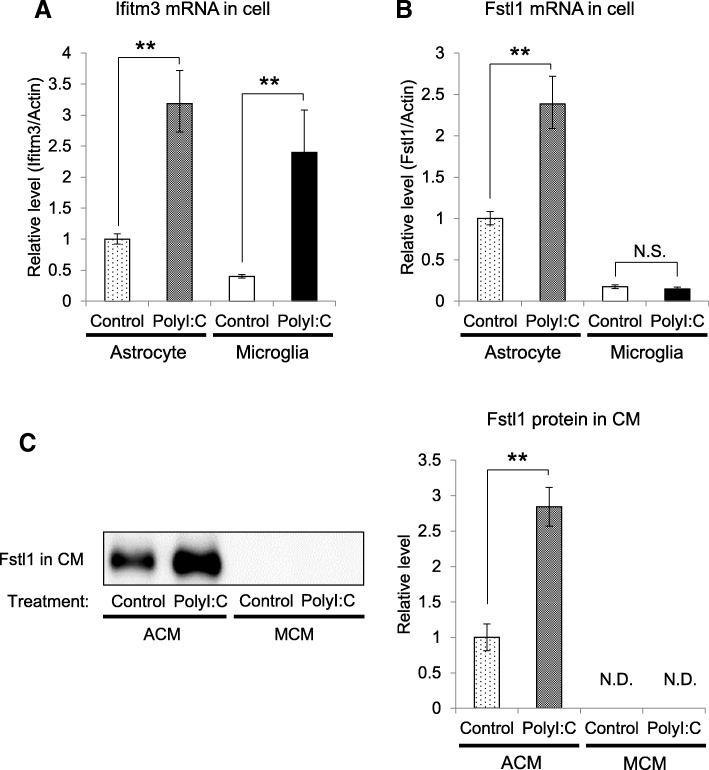
Changes in Ifitm3 and Fstl1 expression in polyI:C-treated astrocytes and microglia. **a** Ifitm3 mRNA levels in polyI:C-treated astrocytes and microglia. Values indicate the means ± SE (*n* = 4). **p < 0.01 versus the respective control treatment. **b** Fstl1 mRNA levels in polyI:C-treated astrocytes and microglia. Values indicate the means ± SE (*n* = 4). N.S., not significant. **p < 0.01 versus control treatment. **c** Fstl1 protein levels in polyI:C ACM and polyI:C MCM. Values indicate the means ± SE (*n* = 7). **p < 0.01 versus control. ACM, astrocyte conditioned medium; MCM, microglia conditioned medium. N.D., not detectable

Because microglia are immunocompetent cells in the CNS, it is possible that microglia also play a similar role in the polyI:C-triggered glial inflammatory response. Accordingly, we examined the mRNA expression of Fstl1 and Ifitm3 in astrocytes and microglia. A two-way ANOVA revealed significant effects of polyI:C treatment on Ifitm3 mRNA levels in astrocyte and microglia cell lysates (polyI:C treatment: *F*(1,12) = 188.0, *p* < 0.01; cell type: *F*(1,12) = 19.91, *p* < 0.01; interaction of polyI:C treatment and cell type: *F*(1,12) = 0.36, *p* = 0.56, Fig. 2a). In contrast, there were significant interactions between cell type and polyI:C treatment on Fstl1 mRNA levels (polyI:C treatment: *F*(1,12) = 25.84, *p* < 0.01; cell type: *F*(1,12) = 127.8, *p* < 0.01; interaction of polyI:C treatment and cell type: *F*(1,12) = 28.04, *p* < 0.01, Fig. 2b). A multiple-comparison test with Bonferroni post hoc tests indicated that polyI:C treatment induced the expression of Fstl1 mRNA in astrocytes, but not in microglia (*p* < 0.01, Fig. 2b). Furthermore, Fstl1 protein was not detected in MCM with or without polyI:C treatment, while Fstl1 protein levels were significantly increased in polyI:C ACM compared to that of the control (*p* < 0.01, Fig. 2c). These results suggest that Fstl1 is induced by polyI:C treatment in astrocytes but not in microglia, and thereby microglia may not play a role in the regulation of extracellular Fstl1 level.

**Fig. 3:**
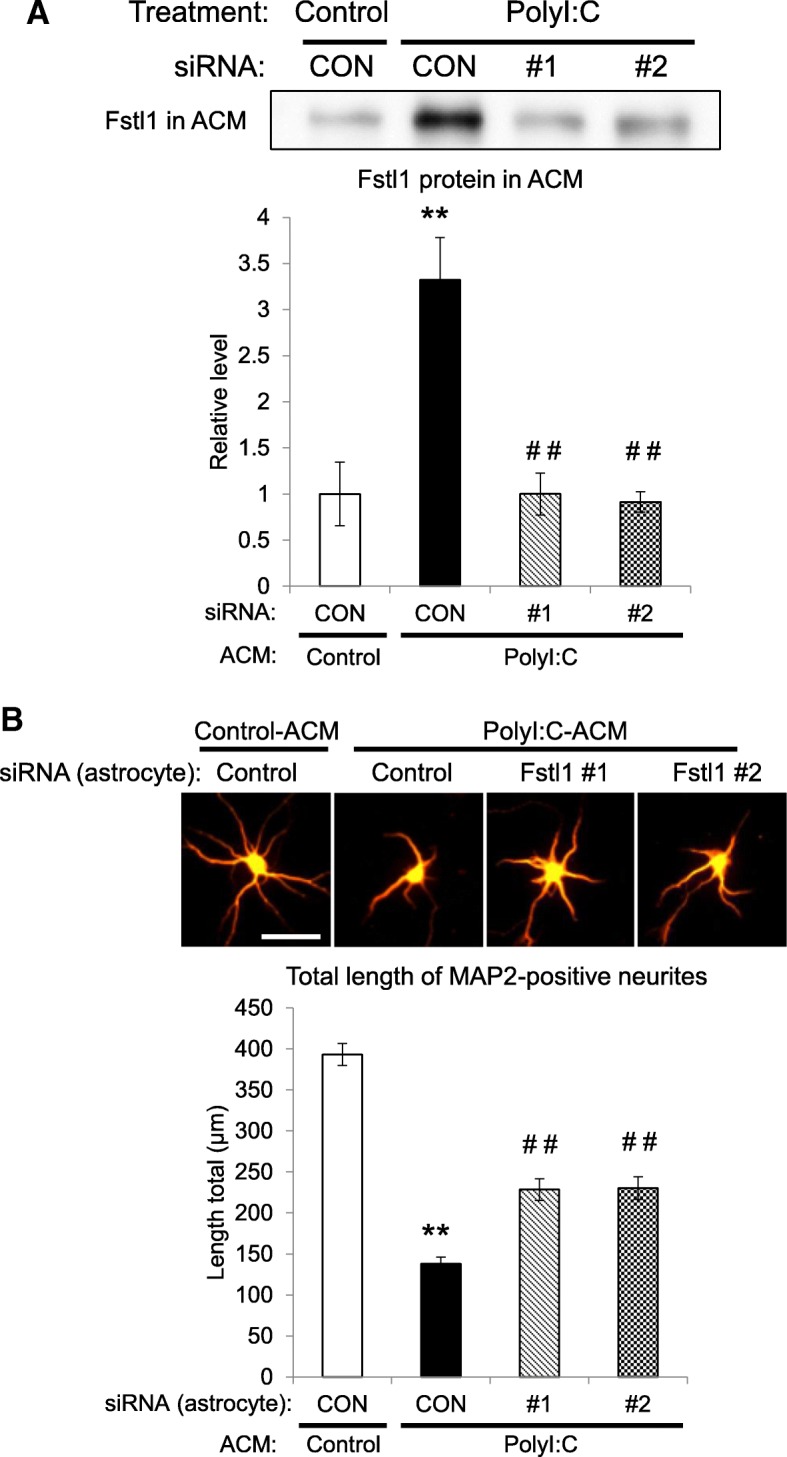
Effect of Fstl1 knockdown in astrocytes on polyI:C ACM-induced impairment of neuronal development. Astrocytes were transfected with control siRNA (CON), Fstl1 siRNA #1 (#1), or Fstl1 siRNA #2 (#2) before polyI:C or vehicle treatment, and then ACM and cell lysate samples were prepared 24 h after polyI:C treatment. **a** Representative western blot images of Fstl1 protein levels in ACM derived from siRNA-treated astrocytes. Values indicate the means ± SE (*n* = 4). **p < 0.01 versus control ACM, ^##^*p* < 0.01 versus control siRNA-treated polyI:C ACM. **b** Effect of polyI:C ACM derived from Fstl1 knockdown astrocytes on MAP2-positive dendrite length of primary cultured neurons (DIV7). Neurons were cultured for 5 days (DIV2-7) with control ACM or polyI:C ACM derived from astrocytes transfected with control siRNA (CON) or Fstl1 siRNA (#1 or #2). Values indicate the means ± SE of more than three independent experiments (*n* = 28–59 neurons). **p < 0.01 versus control ACM, ^##^*p* < 0.01 versus control siRNA-treated polyI:C ACM. Scale bar, 50 μm

We previously found that polyI:C-treated ACM impairs dendritic elongation of cultured hippocampal neurons [17]. Therefore, the role of Fstl1 in dendritic elongation was assessed using an RNA interference method. The effect of siRNA was confirmed by western blotting. When primary cultured astrocytes were transfected with control, Fstl1 #1 or #2 siRNA, the expression levels of Fstl1 in #1 or #2 siRNA were significantly decreased to 34% and 22% of control siRNA transfected cell (Additional file 1: Figure S1). Under this condition, Fstl1 protein levels in ACM were significantly increased by polyI:C treatment (*F*(3,12) = 14.09, *p* < 0.01, Fig. 3a). When astrocytes were transfected with either Fstl1 siRNA #1 or #2 to down-regulate Fstl1 expression, polyI:C failed to increase extracellular Fstl1 protein levels in ACM, and the levels were similar to those in control ACM (*F*(3,12) = 14.09, *p* < 0.01, Fig. 3a). To assess whether Fstl1 plays a negative role in neurite elongation, we measured dendrite length of primary cultured hippocampal neurons which were cultured with the ACM derived from either control or siRNA-treated astrocytes. PolyI:C-treated ACM impaired the dendritic elongation of cultured hippocampal neurons in control siRNA-transfected groups. This impairment was partially attenuated when astrocytes were transfected with either Fstl1 siRNA #1 or #2 (*F*(3,174) = 92.53, *p* < 0.01, Fig. 3b).

**Fig. 4:**
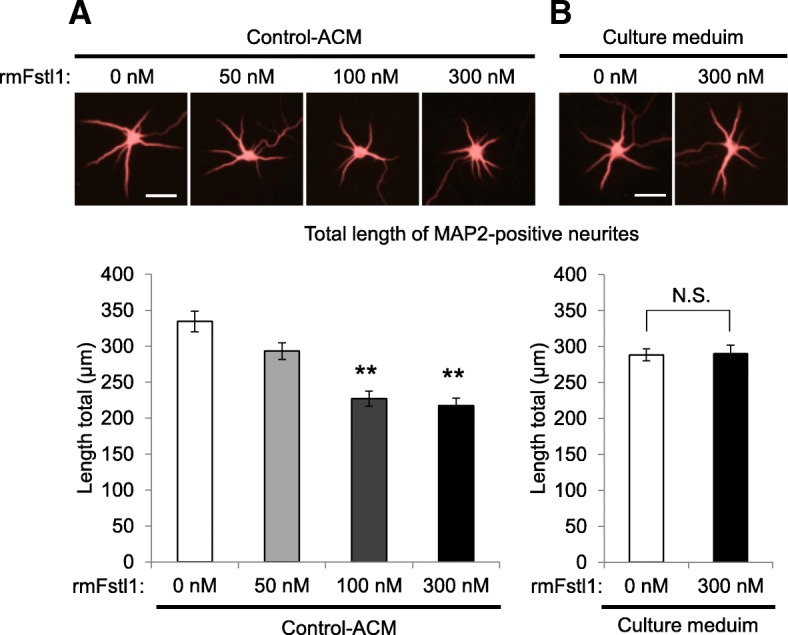
Effect of Fstl1 on dendritic elongation of primary cultured neurons. Neurons were cultured for 5 days (DIV2–7) with control ACM or culture medium supplemented with the indicated concentration of rmFstl1 or vehicle. **a**, **b** MAP2-positive dendrite length of neurons cultured with control ACM (**a**) and culture medium (**b**) supplemented with the indicated doses of rmFstl1 or vehicle (DIV7). Values indicate the means ± SE of three independent experiments (*n* = 49–55 for control ACM and *n* = 52 for culture medium). **p < 0.01 versus vehicle-treated control ACM. Scale bar, 50 μm

We further examined whether Fstl1 protein could mimic the above deleterious effect of polyI:C ACM. Addition of recombinant mouse Fstl1 protein to control ACM on DIV2 resulted in a concentration-dependent decrease in dendrite length of primary cultured neurons assayed on DIV7 (*F*(3,203) = 22.08, *p* < 0.01, Fig. 4a). The decrease in dendrite length induced by rmFstl1 treatment was 12% (50 nM), 32% (100 nM), and 35% (300 nM) compared to that treated with control ACM alone, and a significant decrease of dendritic length was observed at more than 100 nM rmFstl1 (*p* < 0.01, Fig. 4a). Of note, the addition of 300 nM rmFstl1 to normal culture medium on DIV2 had no effect on dendritic elongation of primary cultured hippocampal neurons assayed on DIV7 (Fig. 4b). rmFstl1 treatment had no effect on branched number of neurites and viability of neurons (Additional files 2 and 3: Figures S2 and S3). These results suggest that Fstl1 itself may not directly inhibit the dendritic elongation of neurons but interrupt neurite elongation by cooperating with some factors in ACM.

**Fig. 5:**
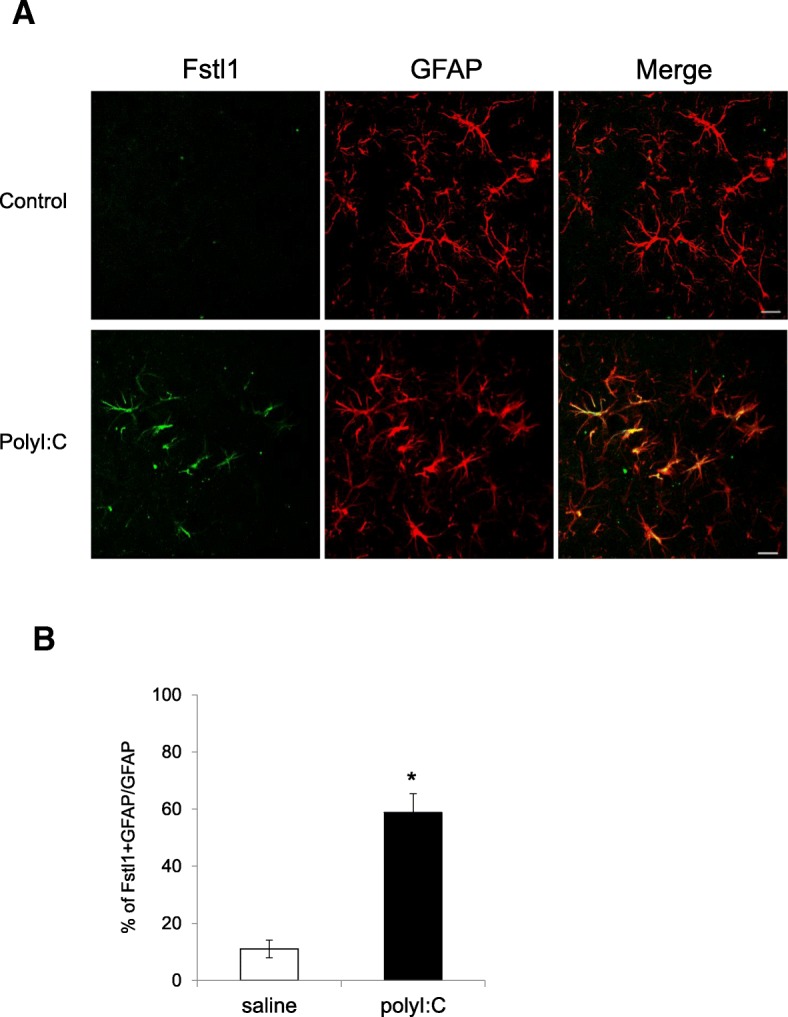
In vivo expression of Fstl1 protein in PD7 neonates. Neonatal C57BL/6J mice were administered with a daily subcutaneous injection of saline (control) or polyI:C (5 mg/kg) between PD2 and PD6. **a** Hippocampal sections were isolated 24 h after the final drug treatment and Fstl1 (green) and GFAP (red, a marker for astrocytes) protein expressions were assessed by IHC. Scale bar, 10 μm. **b** Ratio of Fstl1- and GFAP-double positive cells versus GFAP positive cells was quantified. Values indicate the means ± SE of three independent experiments. *p < 0.05 versus control

Finally, the in vivo expression of Fstl1 in the hippocampus 24 h after neonatal polyI:C treatment was analyzed by IHC. The expression of Fstl1 was hardly detected in vehicle-treated control mice under the same experimental conditions (Fig. 5a). Fstl1 immunofluorescence was clearly observed in the hippocampus of polyI:C-treated mice, and the signal coincided with GFAP, a marker for astrocytes (Fig. 5a). Approximately, 60% of astrocytes were positive for Fstl1 in the hippocampus of polyI:C-treated mice (Fig. 5b). The co-expression of Fstl1 with Iftim3 in astrocyte was observed in the hippocampus of polyI:C-treated neonatal mice (Additional file 4: Figure S4). The expression level of Fstl1 in polyI:C-treated Ifitm3 KO mice was comparable to the level in polyI:C-treated WT mice (Additional file 5: Figure S5).

## Discussion

We have previously demonstrated that mice received neonatal polyI:C treatment exhibit some characteristics of neurodevelopmental disorders including cognitive and emotional impairments in adulthood, which is accompanied by the decrease in spine density and dendrite complexity of pyramidal neurons in the frontal cortex [16, 17, 20]. PolyI:C treatment induces expression of several inflammation-related genes, and induction of these molecules plays a pivotal role in polyI:C-induced neuronal impairment. We have already demonstrated that Ifitm3 expression is increased by polyI:C treatment in astrocytes. Ifitm3 protein localizes to the early endosomes and reduces the endocytic activity of astrocytes, which may change the composition of the extracellular humoral factors. Ifitm3-mediated accumulation of humoral factors released from astrocytes is a determinant in polyI:C-induced neurodevelopmental impairment [17]. Mmp-3 is one of such humoral factors that contribute to the polyI:C-induced neuronal impairment [18].

In the present study, we demonstrated that extracellular Fstl1 level was also dramatically increased in polyI:C-treated astrocytes. We confirmed that Fstl1 is upregulated in polyI:C-treated astrocytes and acts as a humoral factor to impair neurite elongation of cultured neurons. PolyI:C stimulates toll-like receptor 3 (TLR3) signaling, which leads to production of inflammatory cytokines [17, 21], while the expression level of Fstl1 mRNA increases in response to inflammatory cytokines such as IL-1, IL-6, and TNF-α [22, 23]. Therefore, polyI:C may upregulate Fstl1 mRNA levels through the production of inflammatory cytokines.

Fstl1 is a glycoprotein that is secreted in response to inflammatory signals. Lipopolysaccharide (LPS) increases Fstl1 expression [24, 25]. In the present study, we demonstrated that the extracellular level of Fstl1 in cultured astrocytes was increased by polyI:C treatment, but such increase was attenuated in Ifitm3 KO astrocytes. Moreover, co-expression of Fstl1 and Ifitm3 in COS7 cells significantly increased the extracellular level of Fstl1 compared to the level in COS7 cells only expressing Fstl1 but not Ifitm3. We speculate that polyI:C treatment may increase the extracellular level of Fstl1 through the inhibitory effect of Ifitm3 of endocytic activity [17]. An alternative explanation is that Ifitm3 may affect membrane vesicle trafficking and secretion in astrocytes. Because neither the mRNA expression level nor were the intracellular protein level of Fstl1 affected by Ifitm3 expression, regulation of Fstl1 expression may be independent on Ifitm3 expression. Ifitm3-independent expression of Fstl1 was also demonstrated in vivo. Although we attempted to measure extracellular Fstl1 in mice, it has not been detected yet because of analytical limitation. Further study is needed to directly show that the increased extracellular Fstl1 is responsible for the polyI:C-induced neuronal impairment.

In addition to astrocytes, microglia are involved in the inflammation-related neuronal impairment [26, 27]. PolyI:C activates both astrocytes and microglia through activation of TLR3 [28, 29], which led us to hypothesize that Fstl1 expression would be increased not only in astrocytes but also in microglia, in response to polyI:C stimulation. The expression level of Ifitm3 mRNA was significantly increased by treatment with polyI:C both in cultured astrocytes and in cultured microglia. On the other hand, the expression level of Fstl1 mRNA was very limited in microglia compared to astrocytes, and not increased by polyI:C treatment in microglia. Furthermore, the extracellular Fstl1 protein of cultured microglia could not be detected either in the presence or absence of polyI:C treatment. These results suggest that the induction of Fstl1 in response to TLR3 activation by polyI:C may be a specific event in astrocytes, but not occur in microglia.

How does Fstl1 impair morphologic neuronal development? The role of Fstl1 is controversial because some findings suggest that Fstl1 functions as a pro-inflammation cytokine [24, 30–32], but others suggest a role as an anti-inflammatory cytokines [25, 33, 34]. Fstl1 protects cells from apoptosis in heat failure through Fstl1 receptor disco-interacting protein 2 (DIP2), which activates the Akt signaling pathway [35, 36]. Some reports suggest that Fstl1 works as a scavenger by sequestering bone morphogenetic protein (Bmp)-4, which results in blockage of BMP signaling during development [37–39]. The addition of both Fstl1 and ACM suppressed neurite outgrowth, but addition of Fstl1 alone did not. Co-treatment of Fstl1 with Bmp-4 unaffected to neurites elongation (Additional file 6: Figure S6). A possible explanation is that secreted Fstl1 from astrocytes may inhibit signals promoting neurite outgrowth BMP-independent manner. In DRG neurons, Fstl1 impairs neurite elongation through activation of Na/K-ATPase [40]. The similar mechanism might operate in the hippocampal neurons. In this study, we could not address the role of Fstl1 in vivo because of technical limitation; it is hard to manipulate gene expression specifically in astrocytes of neonatal mice. Thus, further studies are needed to disclose the role of Fstl1 in polyI:C-induced neuronal impairment.

The association of Fstl1 with neuropsychiatric disorders is unclear. Recently, a SNP in miR-198, whose expression is mutually exclusive to Fstl1, was found to be associated with schizophrenia [41–43]. We found that the expression of Ifitm3 increased the extracellular level of Fstl1, while other studies reported the increased expression of Ifitm3 in the brains of schizophrenia patients [13–15]. Taken together with our present findings, it is possible that Fstl1 may play a role in the pathophysiology of psychiatric disorders on downstream of Ifitm3.

## Conclusions

From our findings, we conclude that the extracellular level of Fstl1 is regulated by Ifitm3 in astrocytes. However, Fstl1 itself may not directly inhibit the dendritic elongation of neurons but interrupt neurite elongation by cooperating with some factors in ACM.

## Additional files

Additional file 1: **Figure S1.** Validation of Fslt1 knockdown, related to Fig. 3. Culture astrocytes were transfected with siRNA targeting for Fstl1 or control siRNA. Western blotting was performed with indicated antibodies. (PPTX 66 kb)

Additional file 2: **Figure S2.** Effect of rFstl1 treatment on neurite branch, related to Fig. 4. Neurons were treated with indicated concentration of rmFstl1 or vehicle. Branched number of neurites was counted. Values indicate the means ± SE (*n* = 26–36). (PPTX 46 kb)

Additional file 3: **Figure S3.** Effect of rFstl1 treatment on cell viability, related to Fig. 4. Neurons were treated with indicated concentration of rmFstl1 or vehicle. The cell viability of neurons was measured. Values indicate the means ± SE (*n* = 3). (PPTX 43 kb)

Additional file 4: **Figure S4.** Co-expression of Fstl1 with Ifitm3 in the hippocampus of polyI:C-treated neonatal mice, related to Fig. 5. Hippocampal sections prepared from mice treated with vehicle or polyI:C were immunostained with indicated antibodies. Scale bar, 20 μm. (PPTX 470 kb)

Additional file 5: **Figure S5.** Expression of Fstl1 in polyI:C-treated Ifitm3 KO mice, related to Fig. 5. Hippocampal brain slices prepared from vehicle- or polyI:C-treated Ifitm3 KO mice were immunostained with indicated antibodies. Scale bar, 20 μm. (PPTX 23832 kb)

Additional file 6: **Figure S6.** Combinatory treatment of rBmp4 with rFstl1, related to Fig. 4. Neurons were cultured for 5 days (DIV2-7) with culture medium supplemented with the indicated concentration of rmFstl1 and/or rBmp-4. MAP2-positive dendrite length of neurons was measured. Values indicate the means ± SE of three independent experiments. Scale bar, 50 μm. (PPTX 120 kb)

## Abbreviations

ACM: Astrocyte conditioned medium
Bmp-4: Bone morphogenetic protein-4
CNS: Central nervous system
DIP2: Disco-interacting protein 2
Fstl1: Follistatin-like 1
GFAP: Glial fibrillary acidic protein
Ifitm3: Interferon-induced transmembrane protein 3
IHC: Immunohistochemistry
IL-6: Interleukin-6
LPS: Lipopolysaccharide
MCM: Microglia-conditioned medium
Mmp3: Matrix metalloproteinase 3
PolyI:C: Polyriboinosinic-polyribocytidylic acid
TLR3: Toll-like receptor 3
TNF-α: Tumor necrosis factor-α
