# Supplementary figures and images for: Innate immune activation of astrocytes impairs neurodevelopment via upregulation of follistatin-like 1 and interferon-induced transmembrane protein 3
Source: J Neuroinflammation. 2018 Oct 22;15:295. doi: 10.1186/s12974-018-1332-0 (PMC6198367; doi:10.1186/s12974-018-1332-0)

## Slide 1
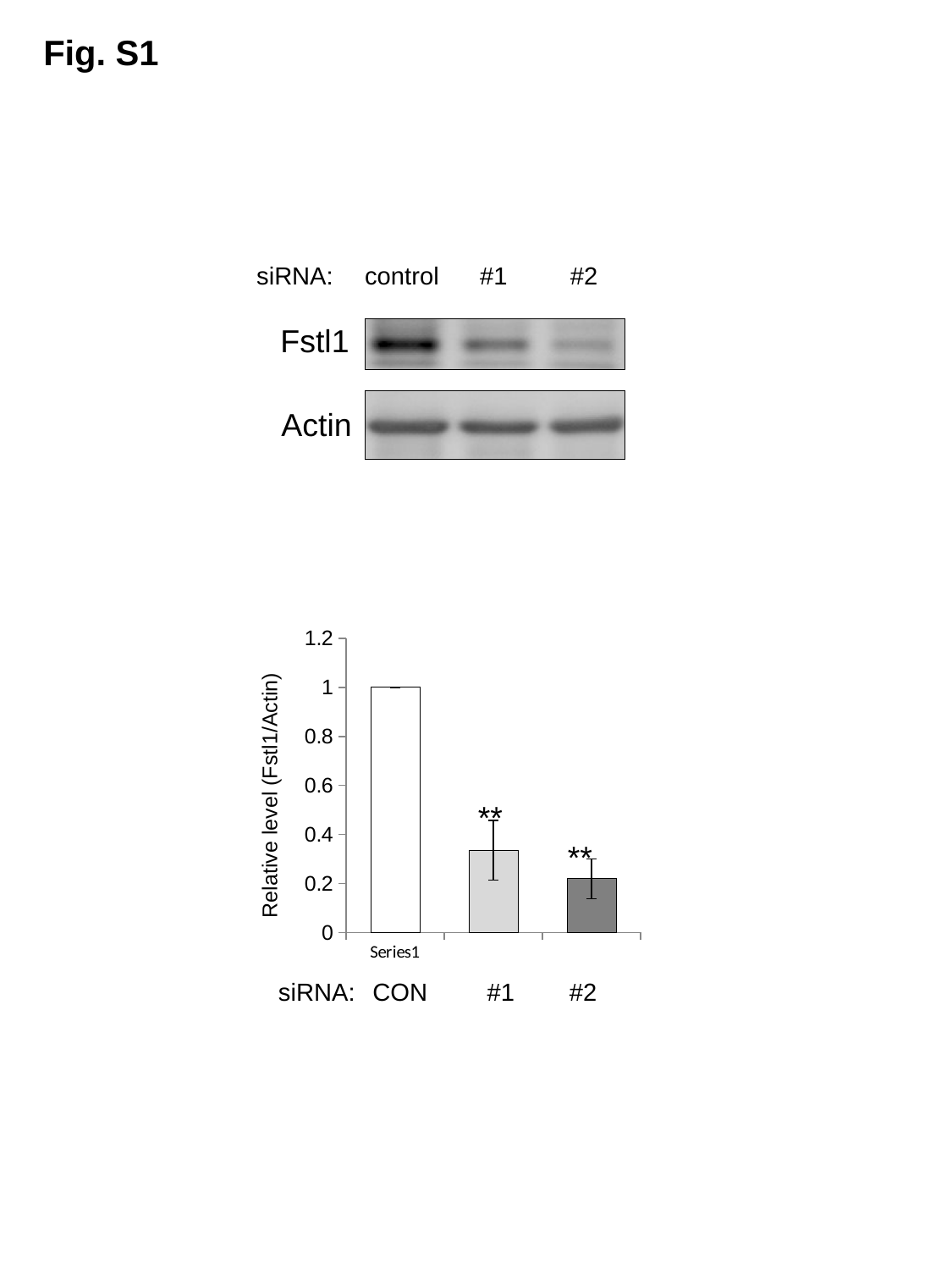

Fig. S1
siRNA:
control
#1
#2
Fstl1
Actin
### Chart
| Category | |
|---|---|
| | 1.0 |
| | 0.335211915719431 |
| | 0.219464241347922 |Relative level (Fstl1/Actin)
**
**
siRNA:
CON
#1
#2

Supplement: Supplementary file 1 — Figure S1. Validation of Fslt1 knockdown, related to Fig. 3. Culture astrocytes were transfected with siRNA targeting for Fstl1 or control siRNA. Western blotting was performed with indicated antibodies. (PPTX 66 kb) [file 12974_2018_1332_MOESM1_ESM.pptx]

## Slide 1
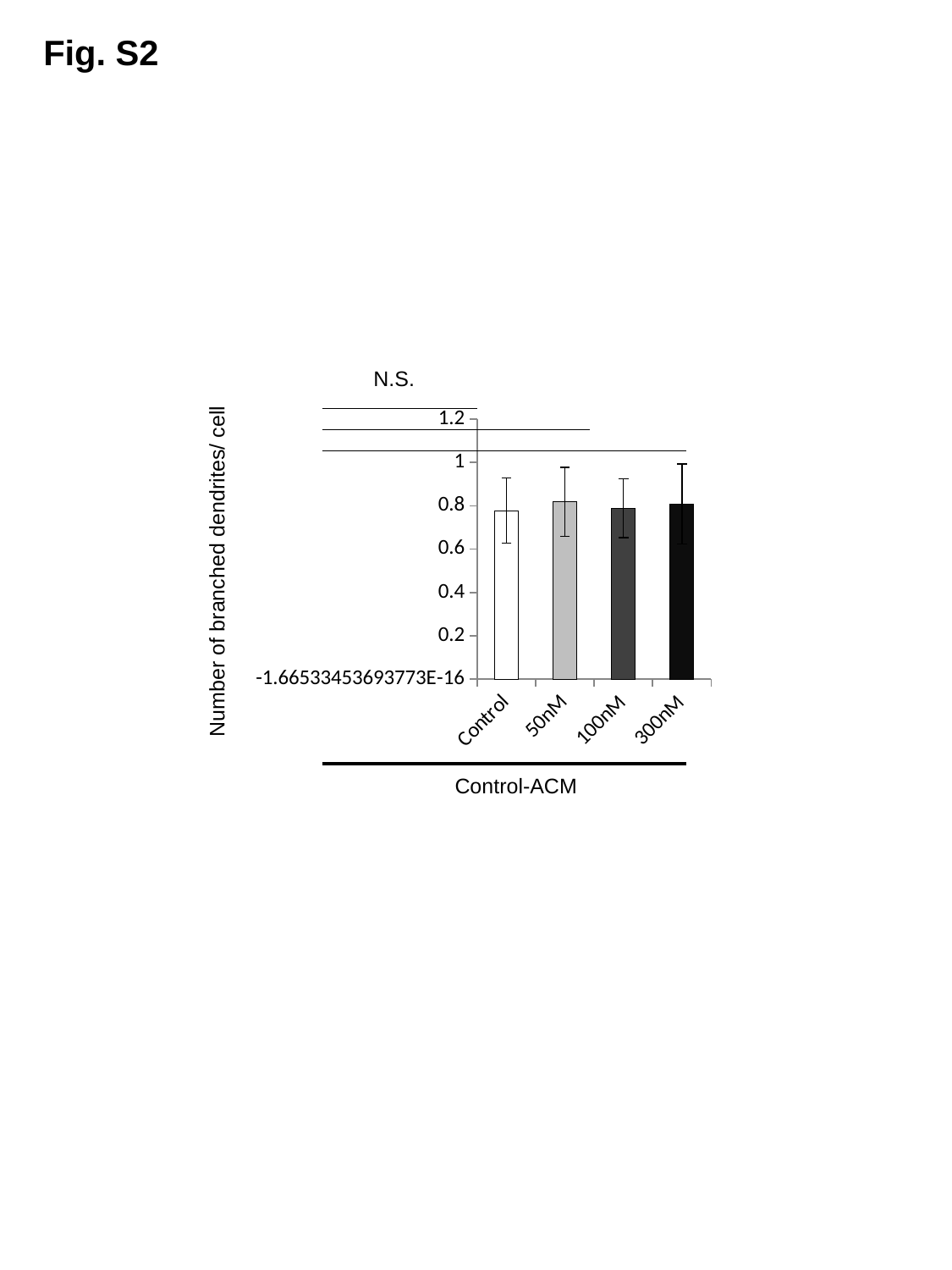

Fig. S2
N.S.
### Chart
| Category | |
|---|---|
| Control | 0.777777777777778 |
| 50nM | 0.818181818181818 |
| 100nM | 0.787878787878788 |
| 300nM | 0.807692307692308 |Number of branched dendrites/ cell
Control-ACM

Supplement: Supplementary file 2 — Figure S2. Effect of rFstl1 treatment on neurite branch, related to Fig. 4. Neurons were treated with indicated concentration of rmFstl1 or vehicle. Branched number of neurites was counted. Values indicate the means ± SE (n = 26–36). (PPTX 46 kb) [file 12974_2018_1332_MOESM2_ESM.pptx]

## Slide 1
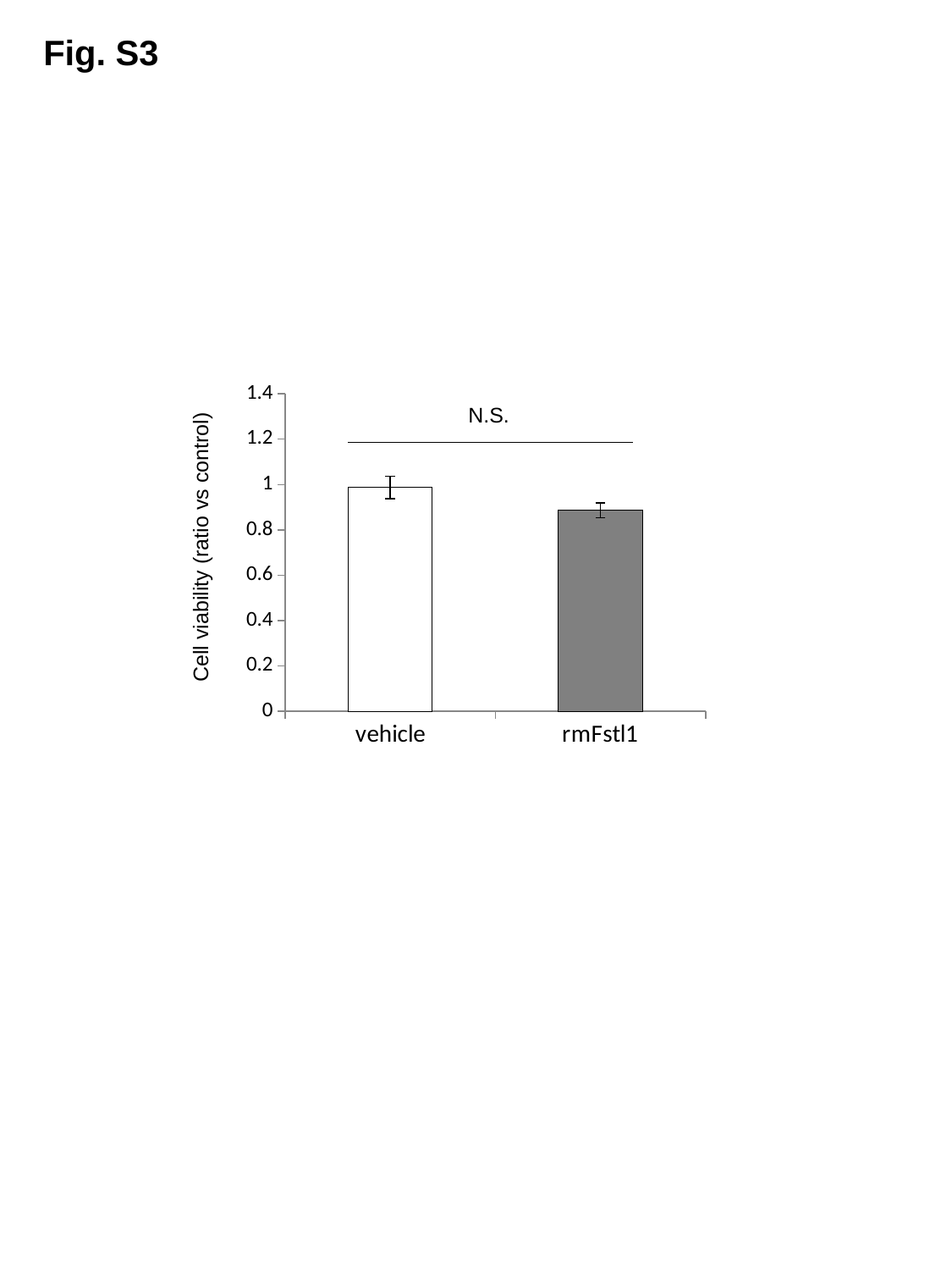

Fig. S3
### Chart
| Category | |
|---|---|
| vehicle | 0.987480438184663 |
| rmFstl1 | 0.885758998435055 |N.S.
Cell viability (ratio vs control)

Supplement: Supplementary file 3 — Figure S3. Effect of rFstl1 treatment on cell viability, related to Fig. 4. Neurons were treated with indicated concentration of rmFstl1 or vehicle. The cell viability of neurons was measured. Values indicate the means ± SE (n = 3). (PPTX 43 kb) [file 12974_2018_1332_MOESM3_ESM.pptx]

## Slide 1
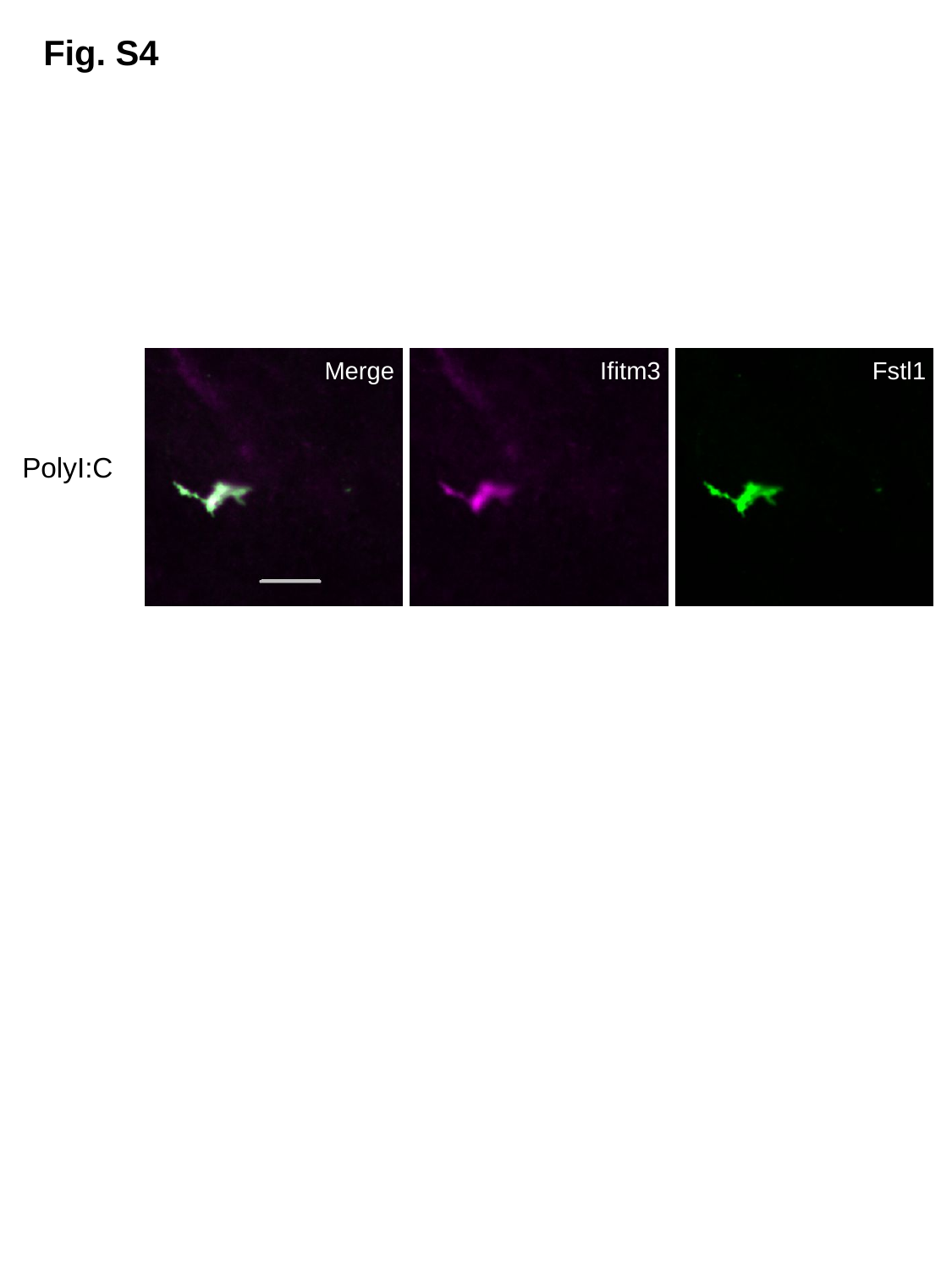

Fig. S4
Merge
Ifitm3
Fstl1
PolyI:C

Supplement: Supplementary file 4 — Figure S4. Co-expression of Fstl1 with Ifitm3 in the hippocampus of polyI:C-treated neonatal mice, related to Fig. 5. Hippocampal sections prepared from mice treated with vehicle or polyI:C were immunostained with indicated antibodies. Scale bar, 20 μm. (PPTX 470 kb) [file 12974_2018_1332_MOESM4_ESM.pptx]

## Slide 1
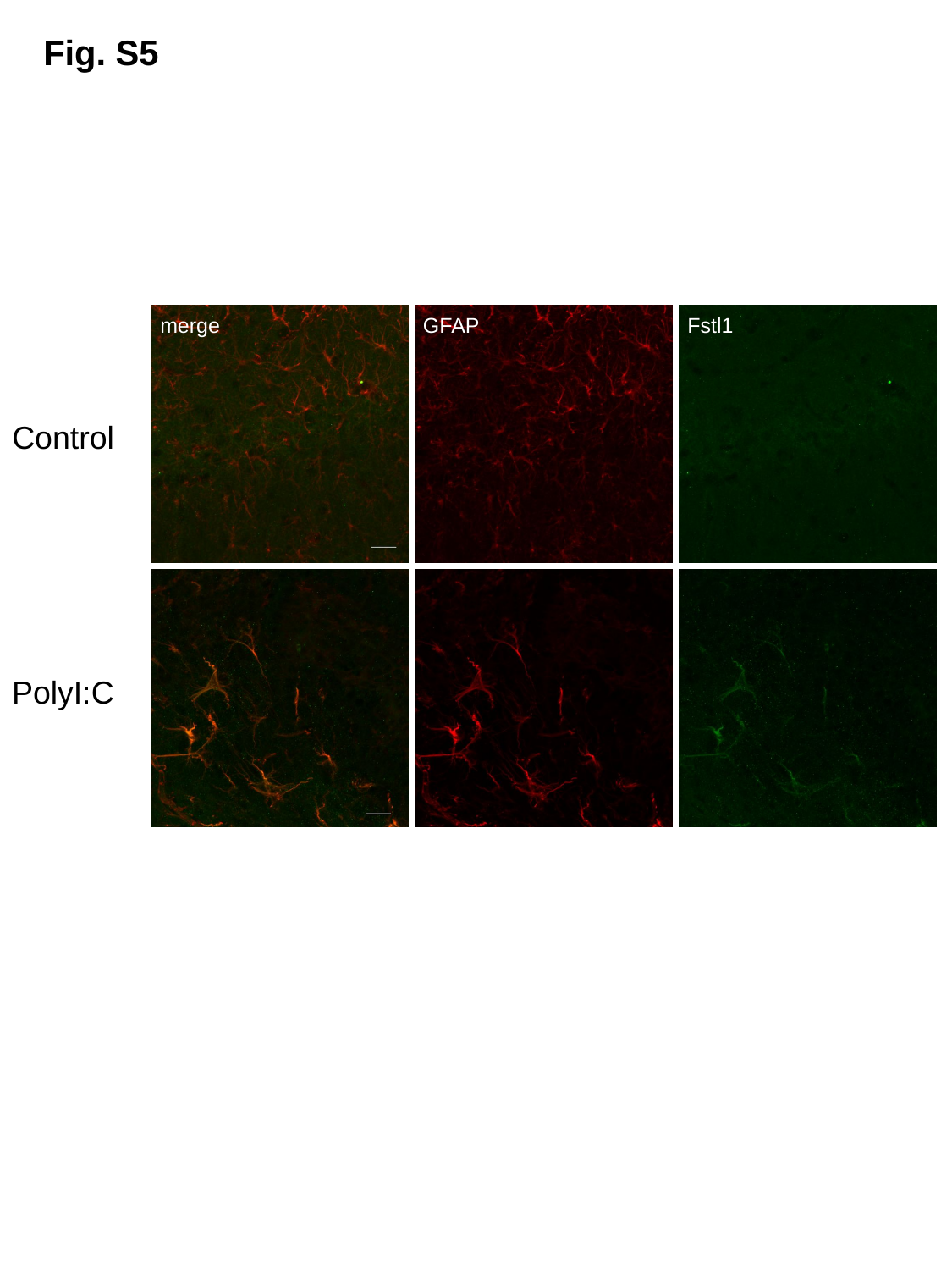

Fig. S5
merge
GFAP
Fstl1
Control
PolyI:C

Supplement: Supplementary file 5 — Figure S5. Expression of Fstl1 in polyI:C-treated Ifitm3 KO mice, related to Fig. 5. Hippocampal brain slices prepared from vehicle- or polyI:C-treated Ifitm3 KO mice were immunostained with indicated antibodies. Scale bar, 20 μm. (PPTX 23832 kb) [file 12974_2018_1332_MOESM5_ESM.pptx]

## Slide 1
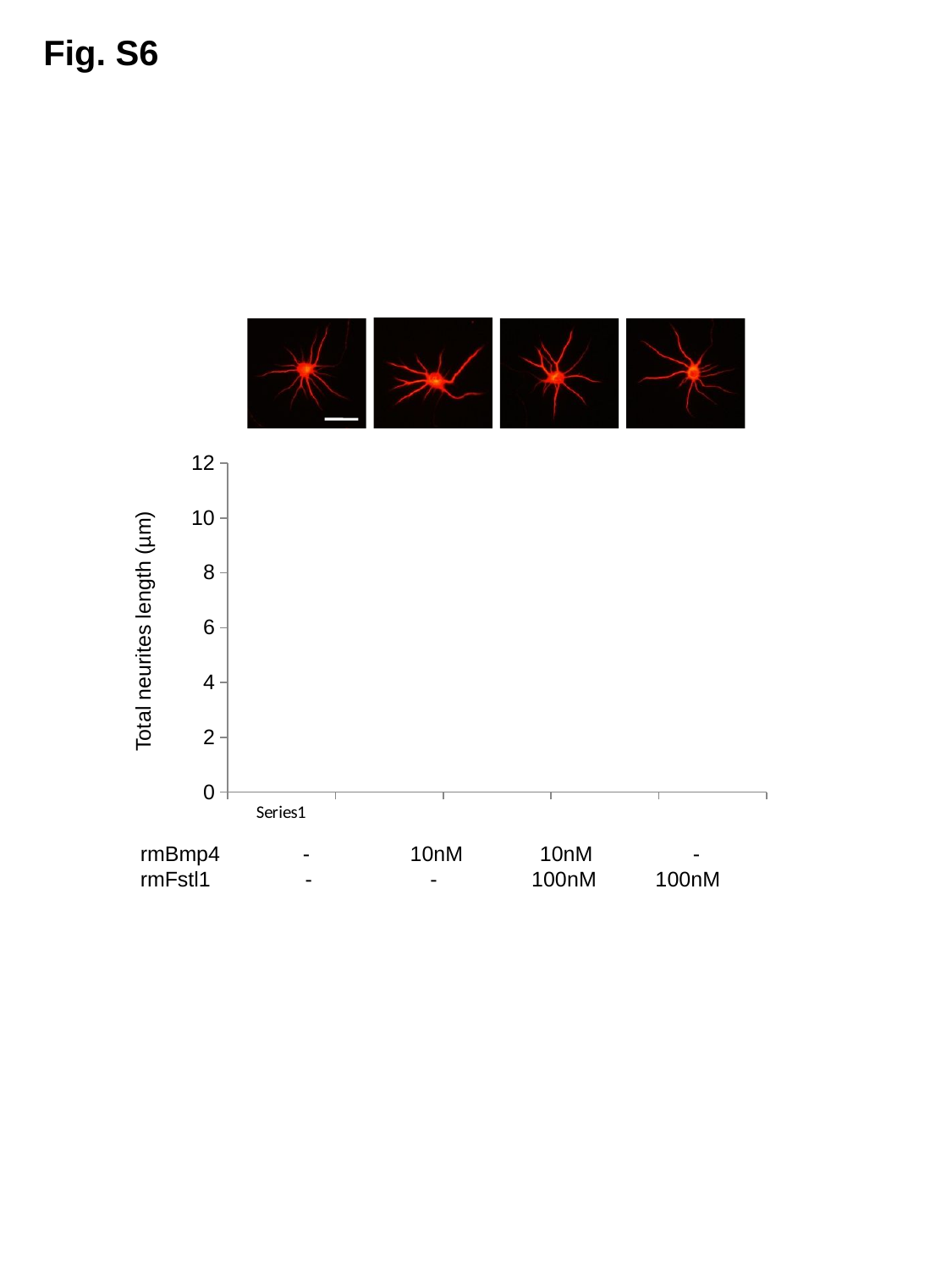

Fig. S6
### Chart
| Category | |
|---|---|
| | 175.3 |
| | 173.9083333333333 |
| | 166.6954545454545 |
| | 163.3428571428572 |Total neurites length (µm)
rmBmp4 - 10nM 10nM -
rmFstl1 - - 100nM 100nM

Supplement: Supplementary file 6 — Figure S6. Combinatory treatment of rBmp4 with rFstl1, related to Fig. 4. Neurons were cultured for 5 days (DIV2-7) with culture medium supplemented with the indicated concentration of rmFstl1 and/or rBmp-4. MAP2-positive dendrite length of neurons was measured. Values indicate the means ± SE of three independent experiments. Scale bar, 50 μm. (PPTX 120 kb) [file 12974_2018_1332_MOESM6_ESM.pptx]
